# Antimicrobial activity of α-(2-hydroxy-2-methylpropyl)-ω-(2-hydroxy-3-methylbut-2-en-1-yl) polymethylene from *caesalpinia bonducella* (L.) Flem

**DOI:** 10.4103/0250-474X.73929

**Published:** 2010

**Authors:** Kavitha Sagar, G. M. Vidyasagar

**Affiliations:** Central Institute of Medicinal and Aromatic Plants, GKVK P.O, Bangalore - 560 065, India; 1 Department of Botany, Gulbarga University, Gulbarga - 585 106, India

**Keywords:** Antibacterial activity, *Caesalpinia bonducella*, pathogenic bacteria, semi arid, yeast

## Abstract

The compound, α-(2-hydroxy-2-methylpropyl)-ω-(2-hydroxy-3-methylbut-2-en-1-yl)polymethylene, isolated from ethyl acetate leaf extract of *Caesalpinia bonducella* (L.) Flem. was evaluated for antimicrobial activity against clinical isolates, *Proteus vulgaris, Pseudomonas aeruginosa, Klebsiella sp., Staphylococcus citrus, Staphylococcus aureus, Escherichia coli, Candida albicans* and *Rhodotorula* sp. using agar diffusion method. The compound exerted inhibitory zone at all concentrations and revealed the concentration-dependent activity against all tested bacterial and yeast strains comparable to standards streptomycin sulphate and gentamycin for bacteria and fluconazole and griseofulvin for *Candida albicans* and Rhodotorula sp. The inhibition zones were wider and clear for *C. albicans* and *Rhodotorula* sp. (IZ >20 mm) and for Pseudomonas aeruginosa, *P. vulgaris* and *E. coli* zones were greater than standards tested, whereas, zones for *Klebsiella* sp. and *S. aureus* were similar to standards.

Plant-derived compounds have been used in different systems of traditional medicine since time immemorial. Interest in plants with antimicrobial properties has revived as a result of current problems associated with the use of antibiotics[1,2] along with the spread of drug-resistant pathogens, which has become one of the most serious threats to successful treatment of microbial diseases. The potential antimicrobial compounds of plants had been related to their ability to synthesize by the secondary metabolism, several chemical compounds of relatively complex structures with antimicrobial activity, including alkaloids, flavonoids, isoflavonoids, tannins, coumarins, glycosides, terpenes, phenylpropannes, organic acids[3]

*Caesalpinia bonducella* (L.) Flem (Fam: Caesalpiniaceae), is a prickly shrub found throughout the tropical parts of India, Myanmar and Sri Lanka. The leaves of this plant are traditionally used for the treatment of tumor, inflammation and liver disorders[4,5]. It has also been recognized for such multiple therapeutic properties that include antipyretic, antidiuretic, anthelmintic, antibacterial[6], anticonvulsant[7], antianaphylactic, antidiarrheal, antiviral[8], antiasthamtic[9], antiinflammatory[10], antiamoebic, antiestrogenic[11], nematocidal[12], antihyperglycemic[13] and abortifacient[14] activities. However, there are no reports on compound isolated from ethyl acetate leaf extract of *C. bonducella* against pathogenic bacteria. Keeping this in view, the present investigation was carried out.

The leaves of *Caesalpinia bonducella* were collected in and around Gulbarga University, Gulbarga during the period from June to December of 2004-2006 and was authenticated at the Herbarium, Department of Botany, Gulbarga University, Gulbarga under voucher No. HGUG- 208. After drying, the material was powdered and subjected to Soxhlet extraction with ethyl acetate solvent. Thirty grams of ethyl acetate leaf extract of *C. bonducella* was chromatographed over silica gel 100-200 mesh on a column of length 52 cm and 6 cm in diameter. Elution was carried out with solvents and solvent mixtures of increasing polarities (0, 10, 20, 30, 40 and 50%). A total of 24 fractions were collected in 250 ml portions. The active 20% (8.0 mg) fraction showed strong antibacterial activity against the test organisms. This fraction was further chromatographed on silica gel column and eluted with hexane:ethyl acetate (20:1)

The column fractions were analyzed by thin-layer chromatography (TLC) (silica gel 60 F_254_, hexane: ethyl acetate, 20:1), and fractions with similar TLC patterns were combined. One fraction showed strong antifungal activity which was finally selected for purification through further TLC. It was found that the fraction showed a single spot on the developed TLC plate. After verifying the purity of an isolated active compound, it was identified based on analysis of its UV, IR, NMR, HPLC and MS spectra. In IR spectrum of the compound the absorption band at 346 cm^-1^ was observed due to the presence of OH group. The absorption peak at 2920 cm^-1^ corresponds to the stretching of C-C bond. The peak that was observed at 1459 cm^-1^ is due to -C=C- stretching. The peak at 1651 cm^-1^ is attributed to due -C-O- stretching. In the ^1^HNMR spectrum the singlet was observed at 0.85 δ due to six protons of two methyl groups attached to C-18. Two singlets at 1.5- and 1.7 δ due to the protons of two methyl groups attached to C-1. The four protons of -2CH_2_- groups present at C-3 and C-17 were resonated as singlet at 2.05 δ and the signals due to of thirteen methylene groups were resonated at 1.1-1.4 δ. The peak due to two -OH groups observed at 5.2 δ as broad singlet. In the mass spectrum, it showed the molecular ion peak at m/z 339 due to [M-1]^+^ ion. This data confirm the structure of the compound as ά-(2-hydroxy-2-methylpropyl)-ω-[2-hydroxy-3-methylbut-2-en-1-yl] polymethylene with the structural formula C_22_H_44_O_2_

Bacterial isolates were generously provided by the Department of Microbiology, Vijayanagara Institute of Medical Sciences, Bellary, Karnataka, India which included *Proteus vulgaris, Pseudomonas aeruginosa, Klebsiella* sp., *Staphylococcus citrus, Staphylococcus aureus, Escherichia coli* and yeast *Candida albicans* and *Rhodotorula* sp. All the organisms were maintained on nutrient agar medium, except *C. albicans* and *Rhodotorula* sp. were cultured on SDA. To obtain cultures, the bacteria were inoculated into the nutrient broth and incubated overnight at 37°. For bioassays, suspension of approximately bacterial cell 1.5×10^6^ CFU/ml in sterile normal saline were prepared as described by Forbes[15]. As a precaution not to miss trace amounts of antimicrobials, a relatively high concentration of 200, 400 and 600 μg/ml of compound was prepared in DMF solvent and administered to fullness in each well. Culture plates were incubated at 37° in case of *Pseudomonas aeruginosa, Candida albicans and Rhodotorula* spp and 27° for remaining test bacteria. A positive control streptomycin sulphate (10 μg/ml), fluconazole (10 μg/ml), griseofulvin 40 μg/ml and gentamycin (40 μg/ml) were used and a negative control DMF was used. After 24 h, bioactivity was determined by measuring diameter of inhibition zones (DIZ) in millimeter. All tests were performed in triplicate. The *in vitro* results were classified as follows: if the compound displayed the inhibition zone less than 10 mm; the antibacterial activity was considered weak ‘a’, if inhibition zone was between 10-15 mm; the antibacterial activity was considered moderate ‘b’; if inhibition zone was between 15-20 mm; the antibacterial activity was considered good ‘c’, if > 20 mm; antibacterial activity was considered strong ‘d’.

The evaluation of the activity of compound against clinical bacterial pathogens and yeast like fungi viz., *Candida albicans* and *Rhodotorula* sp. by using agar diffusion method is reported in Table 1. The compound exerted inhibitory zone at all concentrations and revealed the concentration dependent activity against all tested bacterial and yeast strains comparable to standards, streptomycin sulphate and gentamycin for bacteria and fluconazole and griseofulvin for *Candida albicans* and *Rhodotorula* sp. While assessing antibacterial activity of compound, maximum inhibition was observed against all the tested bacterial strains at all concentrations and the zones were greater than standards with varying magnitudes. The inhibition zones were wider and clear for *C. albicans* and *Rhodotorula* sp. (IZ >20 mm) and for *Pseudomons aeruginosa, P. vulgaris, E. coli* zones were greater than standards tested, whereas, zones for *Klebsiella* sp. and *S. aureus* were similar to standards.

**TABLE 1:** EFFECT OF MICS OF COMPOUND ON TEST BACTERIA AND YEAST LIKE FUNGI

Concn μg/ml	Zone of inhibition (mm)							
	*Ps. a*	*P. v*	*E. c*	*S. c*	*Kl. p*	*S. a*	*Rh*	*C. a*
200	28±0.88^d^	15±0.33^c^	18±0.57^c^	16±0.57^c^	19±0.88^c^	15±0.88^c^	19±0.66^c^	15±0.33^c^
400	34±0.57^d^	17±0.57^c^	21±0.58^d^	17±0.57^c^	19±0.28^c^	20±0.57^d^	23±0.57^d^	19±0.57^c^
600	35±0.57^d^	20±0.51^d^	24±0.51^d^	19±0.88^c^	22±0.57^d^	25±0.57^d^	27±0.57^d^	20±0.88^d^
*	29±0.66^d^	14±0.33^b^	20±0.57^d^	29±0.57^d^	22±0.57^d^	16±0.88^c^	NT	NT
**	32±0.57^d^	17±0.57^c^	23±0.57^d^	38±0.57^d^	22±0.57^d^	22±0.57^d^	NT	NT
***	NT	NT	NT	NT	NT	NT	30±0.57^d^	32±0.57^d^
****	NT	NT	NT	NT	NT	NT	35±0.57^d^	33±0.57^d^

a= weak activity;

b= moderate activity;

c=good activity;

d= strong activity.

Ps. a = Pseudomonas aeruginosa;

P. v = Proteus vulgaris;

E. c = Escherichia coli;

Rh= Rhodotorula spp;

Kl. p = Klebsiella sp.,;

S. a *= Staphylococcus aureus;*

S. c= Staphylococcus citrus;

C. a= Candida albicans.

* Streptomycin 25 μg/ml;

** gentamycin 10 μg/ml;

*** griseofulvin 40 μg/ml;

**** fluconazole 10 μg/ml; Values are mean inhibition zone (mm)±SD of three replicates; P<0.05

NT= Not tested.

Generally, most of the tested organisms were sensitive to the compound and *Pseudomonas aeruginosa, E. coli* and *Klebsiella* sp. were the most susceptible and comparably *S. citrus* was found to be the resistant organism. In the present study, the compound strongly exerted inhibition against *S. aureus* and *Pseudomonas aeruginosa* of clinical origin, which are spreading hazards in the world. The strong activity of the compound may be due to its easily diffusible nature that permits to enter the cell wall of tested bacteria in the study without any permeable barriers. Despite the fact that standard antibiotics used in this study were found to be superior to the compound, it still showed moderate, but constant activity against all the tested bacterial strains and yeast *Candida albicans* and *Rhodotorula* of clinical origin.

Since, there is no scientific evidence to support the medical use of ά-(2-hydroxy-2-methylpropyl)-ω-[2-hydroxy-3-methylbut-2-en-1-yl] polymethylene, further studies are needed in order to elucidate the mechanism(s) of action of this compound, as well as the antimicrobial activity against other microbial strain in particularly antibiotic resistant bacteria. Further, detailed study of cellular alterations and biochemical studies in the compound treated bacteria is also recommended which provide the potentiality of the compound.
